# Scale-Dependent Habitat Selection and Size-Based Dominance in Adult Male American Alligators

**DOI:** 10.1371/journal.pone.0161814

**Published:** 2016-09-02

**Authors:** Bradley A. Strickland, Francisco J. Vilella, Jerrold L. Belant

**Affiliations:** 1 Department of Wildlife, Fisheries, and Aquaculture, Mississippi State University, Mississippi State, Mississippi, United States of America; 2 U.S. Geological Survey, Mississippi Cooperative Fish and Wildlife Research Unit, Mississippi State University, Mississippi State, Mississippi, United States of America; 3 Carnivore Ecology Laboratory, Forest and Wildlife Research Center, Mississippi State University, Mississippi State, Mississippi, United States of America; Auburn University, UNITED STATES

## Abstract

Habitat selection is an active behavioral process that may vary across spatial and temporal scales. Animals choose an area of primary utilization (i.e., home range) then make decisions focused on resource needs within patches. Dominance may affect the spatial distribution of conspecifics and concomitant habitat selection. Size-dependent social dominance hierarchies have been documented in captive alligators, but evidence is lacking from wild populations. We studied habitat selection for adult male American alligators (*Alligator mississippiensis*; *n* = 17) on the Pearl River in central Mississippi, USA, to test whether habitat selection was scale-dependent and individual resource selectivity was a function of conspecific body size. We used K-select analysis to quantify selection at the home range scale and patches within the home range to determine selection congruency and important habitat variables. In addition, we used linear models to determine if body size was related to selection patterns and strengths. Our results indicated habitat selection of adult male alligators was a scale-dependent process. Alligators demonstrated greater overall selection for habitat variables at the patch level and less at the home range level, suggesting resources may not be limited when selecting a home range for animals in our study area. Further, diurnal habitat selection patterns may depend on thermoregulatory needs. There was no relationship between resource selection or home range size and body size, suggesting size-dependent dominance hierarchies may not have influenced alligator resource selection or space use in our sample. Though apparent habitat suitability and low alligator density did not manifest in an observed dominance hierarchy, we hypothesize that a change in either could increase intraspecific interactions, facilitating a dominance hierarchy. Due to the broad and diverse ecological roles of alligators, understanding the factors that influence their social dominance and space use can provide great insight into their functional role in the ecosystem.

## Introduction

Wildlife management and conservation frequently rely on understanding mechanisms that influence the spatial distribution of organisms [1]. Animals distribute themselves in the environment by searching for the most suitable areas to obtain food, access to mates, and other resources that will optimize allocation to reproduction and survivorship, and consequently, maximize fitness [2,3]. Habitat selection is an active behavioral process that may vary across spatial and temporal scales [4,5]. Animals choose an area of primary utilization (i.e., home range) then make decisions focused on resource needs within patches in the area [4,5]. Therefore, larger spatial patterns may determine the selection of an animal’s home range, whereas resources such as food are selected at finer scales [6]. Limiting factors may often drive behavior and influence selection at the coarsest spatial scales (e.g., population, home range) in order to maximize individual fitness [7]. Therefore, habitat selection may vary across scales and involve innate and learned behaviors [8]. This makes the selection process complex and influenced by more than just resource availability, including factors such as predation and competition [9].

One behavioral mechanism, dominance, may affect the spatial distribution of conspecifics [2,3]. Despotism, through dominance hierarchies or territoriality, arises when competitive asymmetry constrains the ability for any individual to occupy all areas within the habitat, resulting in spatial segregation and exclusion of subordinates from suitable resources [10–12]. Dominance in animal social structures is manifested through traits including age, gender, body size, and aggression [13]. Further, dominance rank can purportedly influence an individual’s reproductive success, survivorship, foraging efficiency, and may also influence movements and resource selection [13,14]. Crocodilian social behavior has been characterized by size-dependent absolute hierarchies (independent of time and location), where large males control access to mates and food [15–18]. This social structure has been observed in captive American alligators (*Alligator mississippiensis*) [19,20], but evidence is lacking for wild populations. Social dominance in crocodilians appears to be asserted by species-specific, complex social signals (e.g., exposing body length and inflating posture) [17]. Social signals such as head-slapping and bellowing are observed frequently in adult male alligators during the breeding season and may serve to define territory, claim mates, and establish dominance [19].

Understanding the mechanisms that drive habitat selection of crocodilians is valuable given their role as top predators in aquatic ecosystems may influence lower trophic levels through top-down effects [21–24]. Our objective was to examine space use of a wild population of male American alligators in an inland riverine system to determine habitat selection patterns and secondarily, to infer the role of body size on selection patterns in the context of dominance hierarchies. We studied habitat selection at coarse (home range) and fine (patch) scales to determine if congruency in selection existed and what habitat variables are important for selection. We tested two alternative hypotheses: 1) habitat selection is consistent across spatial scales or 2) habitat selection is hierarchical and varies across scales. Overall, we predicted no congruency in selection between scales because one general habitat selected at the coarse scale is unlikely to satisfy the multiple needs (e.g., thermoregulation, foraging) that may be more important in finer scale selection [5]. Further, if a dominance hierarchy was present we predict that larger, dominant alligators will control access to suitable resources, and individual resource selectivity would be a function of conspecific body size at both scales [3], particularly, for home range selection [25]. We predicted selection for and proximity to areas of suitable habitat (i.e., deep, open water [26,27]) within home ranges will increase with increasing body size. Animals should occupy the smallest area needed to acquire resources to maximize individual fitness; however the mechanisms that influence intraspecific variation in home range size are poorly understood [3,28]. Thus, we predicted that dominant individuals will have smaller home ranges (despite larger energetic needs) because they occupy more suitable habitat.

## Study Area

**Fig 1:**
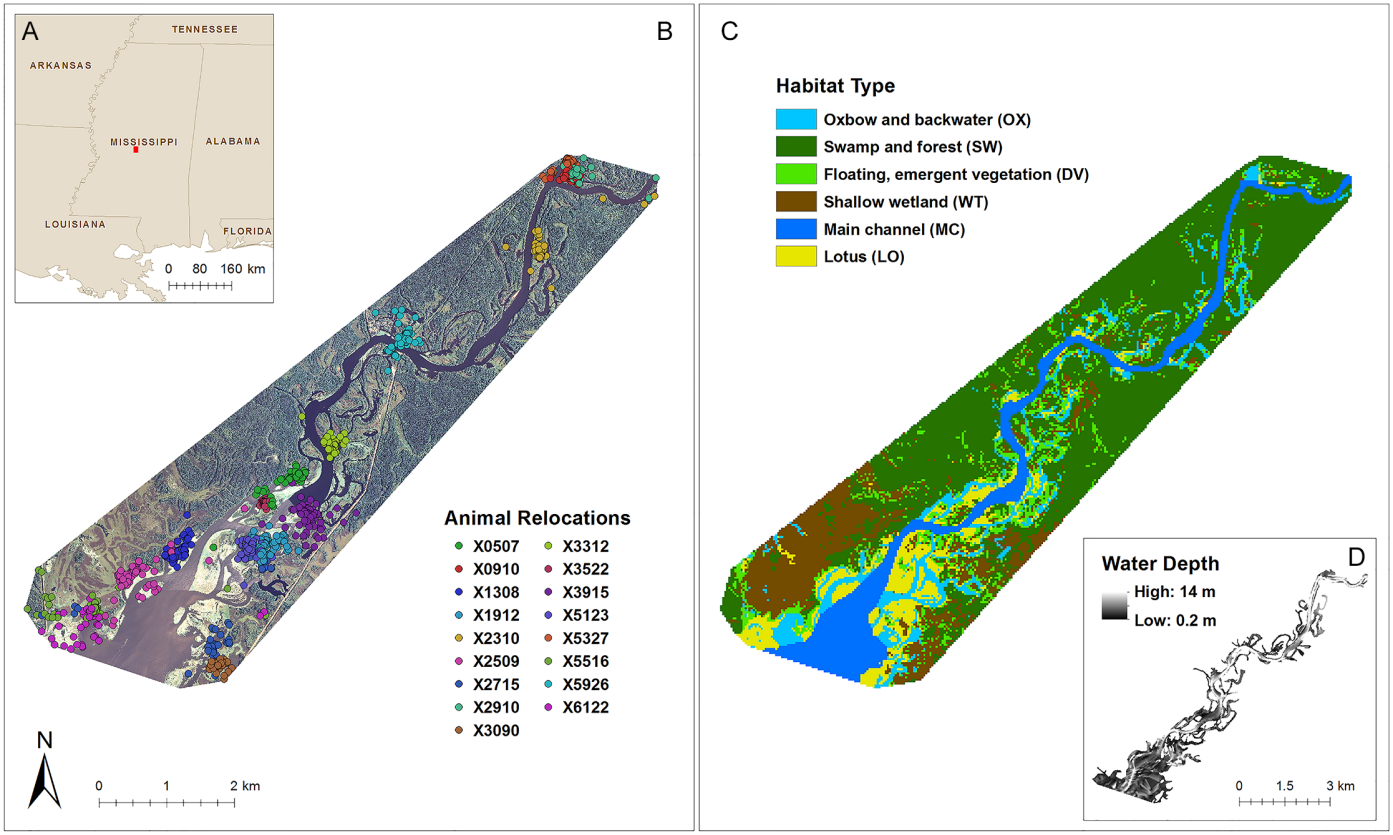
Map of the study area and radio-tracked adult male alligator relocations at Ross Barnett Reservoir and the Pearl River, Mississippi, USA, 2012–2013. (A) Inset shows study area as a solid red square. (B) Aerial imagery with animal relocations for 2012 and 2013 combined. (C) Categorical map of the six habitat types classified from aerial imagery. (D) Bathometric display of study area. Outside the main channel and reservoir white represents “no data” and inside it represents the deepest water.

We conducted the study on the Pearl River and the upper portion of the Ross Barnett Reservoir (RBR) in central Mississippi (32°31’12.3”N 89°55’28.1”W; Fig 1A and 1B). The Pearl River is a sand- and gravel-bottomed river that flows southwest to the Mississippi Sound draining 23,000 km^2^ of bottomland forest and agricultural lands [29]. The RBR is a 133.5 km^2^ monomictic, mesotrophic reservoir created from an impoundment of the Pearl River in 1964 to meet the water supply needs and provide recreational opportunities for the city of Jackson, Mississippi, and surrounding counties [29,30]. The reservoir has a 3.66 m mean depth (10.67 m maximum) where annual water level fluctuations average less than 1 m and few littoral areas become dewatered at any point during the year [30].

## Methods

### Capture and tagging

In spring and summer 2012 we captured adult male alligators using rod and reel assisted by the Mississippi Department of Wildlife, Fisheries, and Parks agent trappers. We attached a weighted (57 g lead) 10/0 treble hook to 68 kg test braided, hi-vis yellow line via a Palomar knot and cast it with a heavy duty baitcast reel and a 2.4 m medium-heavy action rod to snag alligators from a distance. We used a 3.2 mm restraining line, 1.5 m locking cable snare, and 12.7 mm cotton rope (4.5 m in length) to hoist and restrain animals. We then used electrical tape and 6.4 mm nylon rope to secure the mouth and tie legs of captured alligators. For each captured alligator, we recorded gender from cloaca examination [31] and measured total length (TL) from the tip of the snout to the end of the tail following the dorsal contour. We attached a numbered livestock tag (Duflex #6341 and #6321; Destron Fearing; South St. Paul, Minnesota, USA) visible from either side of the tail in the second posterior scute after the scutes merged into a single row and a small metal clip tag (1005–3 or 1005–681 (size 3); National Band & Tag Co.; Newport, Kentucky, USA) in the webbing of each hind foot between the second and third toe.

We fitted adult male alligators (TL > 1.83 m) [31] with a VHF radio-transmitter (Ultimate V5H 227A; Sirtrack; North Liberty, Iowa, USA) by first cleaning the scutes with a disinfectant solution, then drilling a 3.2 mm hole into each of the last 4 tail scutes before the scutes merge into a single row. We then threaded stainless steel wire (0.14 mm diameter) encased in nylon tubing (3.175 mm diameter) through the transmitter and into the holes made in the 4 scutes. Once the transmitter was fitted between the rows of scutes, we inserted the wire through the scutes and the transmitter. We continued threading the same wire through the other paired scutes and tied the ends of the single wire. To reduce potential loss of the transmitter, we covered exposed wire and transmitter with marine-grade epoxy (PC-11; PC-Products; Allentown, Pennsylvania, USA). Finally, we released all animals at the capture site. Mississippi Department of Wildlife, Fisheries, and Parks granted permission and supervised all animal capture and handling. Animal capture and handling procedures were conducted under the auspices of Mississippi State University Institutional Animal Care and Use Committee protocol 12–016. All research was performed on public waterways using public access ramps.

### Radio-telemetry

Before tracking marked alligators, we assessed performance of the radio-transmitters. Specifically, we estimated distance of radio-transmitters to an observer at a specific receiver gain across a range of signal strengths [32]. First, we attached transmitters to channel markers in open water (*n* = 6) about 5–10 cm below the surface and within representative vegetation (*n* = 5) to assess signal attenuation and reflection. We oriented the antenna toward the transmitter and recorded receiver signal strength at each gain level (215 to 235 units in 5-unit increments) from 600 to 25 m at increments of 100, 50, or 25 m. We measured distances using a laser range finder (Elite 4200; Bushnell; Overland Park, Kansas, USA) and verified these distances using a hand-held GPS. Using these data, we conducted generalized linear models to predict distance at a given signal strength and gain. We ran a null model and models including all combinations of signal strength and gain as well as interaction and quadratic terms. We used Akaike’s Information Criterion corrected for small sample sizes (AICc) for model selection [33].

We conducted additional trials to estimate angular and location error of telemetry signals, and determine bias and sampling error [32,34]. One observer placed transmitters (*n* = 10; 4–7 locations each) in open water or locations representing the range of vegetation conditions about 5–10 cm under the surface of the water. Another observer, without prior knowledge of transmitter location, estimated direction to the transmitter using the strongest signal. Bearing, signal strength, gain, and location were recorded at about 2-minute intervals from the time the signal was heard until it was found. We used this information to generate an estimated distance from the transmitter predicted by the best linear model from the previous trial. We compared these distances and bearings to distances and directions verified using GPS to yield location and angular errors. Finally, we used Student’s *t*-tests to compare mean errors to 0 to estimate bias.

We searched for adult male alligators from 1 March to 15 October 2012–2013 from sunrise to sunset; tracking dates correspond with the period of greatest alligator activity at this latitude [26,35,36]. We limited radio-tracking activities to daylight hours and avoided days with inclement weather for personnel safety. A tracking route with each animal’s expected location was generated using each individual’s capture site or recent locations. We started tracking each day at random start times (between 0600 and 1300 hours) allowing enough time to locate all individuals. We selected the first tracked individual using a random number generator. If we did not observe a radio-marked alligator at its expected location, we searched for it in the vicinity and along the tracking route, while continuing to locate other animals. Typically (about 90% of tracking days), all animals were searched for in one daily effort. There were a few instances of animals not being found after a complete search of the study area until days or weeks later. In these cases, we searched for all animals throughout the study period. We randomly selected the direction of tracking along the route daily and order of animals on the tracking route every three months.

We performed radio-telemetry from a boat by homing to a specific transmitter using a VHF digital receiver (TR-5; Telonics; Mesa, Arizona, USA) and a 3-element Yagi antenna (Sirtrack; North Liberty, Iowa, USA). It was frequently impractical or impossible to pinpoint an animal’s exact location due to thick emergent aquatic vegetation or shallow water. In these cases, we approached the radio-marked alligator and homed on the direction of the “strongest signal” [34], taking a directional bearing using a magnetic compass (KB-14; Suunto; Vantaa, Finland), while attempting to minimize disturbing the animal.

### Spatial database development and resource variables

We obtained aerial imagery flown in August 2012 from the United States Department of Agriculture’s National Agriculture Imagery Program (Fig 1B; 1-m resolution, natural color spectral resolution) [37]. We clipped the raster dataset to the study area using a minimum convex polygon (MCP) of all relocations plus a 30 m buffer to account for radio-telemetry error. We also obtained a water depth grid of the study area with 8-m resolution collected by the Mississippi Department of Wildlife, Fisheries, and Parks (1505 Eastover Drive, Jackson, Mississippi) in August 2005 (Fig 1D). We performed all geospatial analyses and resource variables map development using ArcMap 10.2 (Environmental Systems Research Institute; Redlands, California, USA).

Point intercept survey data collected by Sartain et al. (2013) [38] in June 2012 provided detailed accounts of littoral zone (depths < 3 m) plant species at RBR. We overlaid the 300 m grid point intercept survey on the aerial imagery to identify, group, and validate pixel classification. Map pixels were categorized into 6 ecologically relevant habitats using maximum likelihood classification techniques based on visual color and reflectance signatures of the image (Fig 1C) following Cox and Madsen (2011) [39]. Habitat categories included:

1. Main channel and reservoir open water (MC)–Boat and watercraft traffic was common through most areas of the main channel and reservoir.
2. Open water in backwater areas and oxbow lakes (OX)–Backwaters and oxbow lakes were dominated by different plant and animal assemblages than the main channel and are considered important habitats for fish reproduction and recruitment [40,41].
3. Forests and swamps (SW)–This habitat included forested riparian areas along the main river channel, forested islands, backwater swamps, and surrounding oxbow lakes. Black willow (*Salix nigra*) and oak (*Quercus* spp.) dominated these areas and were interspersed with stands of pine (*Pinus* spp.). Alluvial swamps were characterized by bottomland hardwood trees including: bald cypress (*Taxodium distichum*), water tupelo (*Nyssa aquatica*), tupelo gum (*Nyssa sylvatica*), sugarberry (*Celtis laevigata*), elm (*Ulmus* spp.), and hickory (*Carya* spp.) [42].
4. Shallow water wetlands (WT)–This habitat contained sediment and submersed vegetation dominated by native coontail (*Cerotophyllum demersum*) and exotic hydrilla (*Hydrilla verticillata*). Shallow wetlands were typically along the reservoir perimeter.
5. Floating, emergent vegetation (DV)–This habitat included dense mats of exotic alligator weed (*Alternanthera philoxeroides*) and water hyacinth (*Eichhornia crassipes*), native floating plants such as water primrose (*Ludwigia peploides*) and white waterlily (*Nymphatea odorata*), and thick concentrations of native grasses including giant cutgrass (*Zizaniopsis miliacea*) and cattail (*Typha* spp.).
6. Lotus (LO)–This habitat contained highly monotypic populations of American lotus (*Nelumbo lutea*), the most common littoral plant in RBR [38].

To accommodate location error, we resampled the classified raster to 30-m resolution containing the dominant habitat in each 30 m pixel. We also calculated Euclidean distances from the center of each cell to the main channel and nearest floating, emergent vegetation. We created a 30 m point grid (points as centroids of 30 m pixels) over the study site and assigned each grid point a single habitat class (binary output), distance-to-habitat, water depth, and geographic coordinates. Ground-truthing surveys of 25 samples from each habitat yielded a 94% accuracy rate (aggregate metric including errors of both omission and commission).

### Resource selection analysis

We used K-select analysis to estimate individual selection strategies on 17 radio-marked individuals with greater than 15 relocations per year [43]. This method allows evaluation of potentially correlated environmental variables that contribute most to habitat selection by generating marginality vectors for each animal that point from the from the centroid of available resource space to the centroid of used resource space. Vector direction indicates which variables are selected and vector length (squared distance) represents strength of selection by an animal; thus, differences in marginality vectors represent individual variation in selectivity of habitat. [43]. K-select analysis uses a non-centered principal component analysis (PCA) of a table of marginality vectors of each animal (row) on the habitat variables (column) to reduce the multidimensional resource space to principle components (factorial axes), each representing a linear combination of the original habitat variables, while maximizing marginality on the first axis [43,44]. Biological significance of the factorial axes can be inferred from vector loadings of each environmental variable [43,44].

We analyzed second-order (home range level) habitat selection [4] where available habitat was defined by the MCP encompassing all locations in the study area and used habitat was represented by relocations within individual 95% MCP home ranges. We also analyzed third-order (patch level) selection of adult male alligators with available resource space characterized as the 95% MCP home range and used resource space determined from relocations of each animal. Analyses at both scales included a multiannual home range and pooled locations from both years due to a limited sample size of locations for each individual in each year. We performed randomization tests (*n* = 10,000 steps) using the first eigenvalues to determine influence of each habitat variable on marginality of each animal and if observed use differed significantly than what is expected under a random habitat use hypothesis. For these tests, we expected the explanatory variables to vary greatly so we set α = 0.10 using Bonferroni correction. We also extracted the distances of the marginality vectors (measure of selectivity) for each animal and modeled them with body size and home range size as dependent variables using linear regression.

We performed statistical analyses using R (Mac version 3.0.2; R Foundation for Statistical Computing; Vienna, Austria), including the adehabitat package [44], to estimate home ranges and habitat selection. We reported means with ± 1 standard deviation (SD) and outliers were determined using interquartile range [45].

## Results

We captured 20 adult male alligators and used 17 animals with sufficient numbers of relocations for resource selection analyses (S1 Table). We collected on average 34 ± 8 (SD) locations for each animal per active period (approximately 4 to 5 locations per month per animal) totaling 1,145 relocations (Fig 1B). Estimated animal locations were best explained by the equation: distance = -1,765 + 3.523*signal + 9.138*gain—0.0201*signal*gain (adjusted *R*^2^ = 0.684, *F*_[3, 224]_ = 35.71, *P* < 0.001). Mean angular error (-2.4 ± 13.3 degrees) did not differ from zero and directional bearings were unbiased (t_50_ = -1.304, *P* = 0.198; 95% CI = -6.155–1.310). Similarly, location error (t_21_ = 1.126, *P* = 0.2731; 95% CI -5.216–17.523) was unbiased and averaged 6 ± 14 m.

Forests and swamps accounted for 48% of the study area, followed by shallow wetlands (15%), main channel (12%), floating, emergent vegetation (9%), backwaters and oxbow lakes (8%), and lotus (8%). However, percentage of alligator relocations were 24% in forests and swamps, shallow wetlands (19%), main channel (2%), floating emergent vegetation (29%), backwaters and oxbow lakes (14%), and lotus (12%).

**Fig 2:**
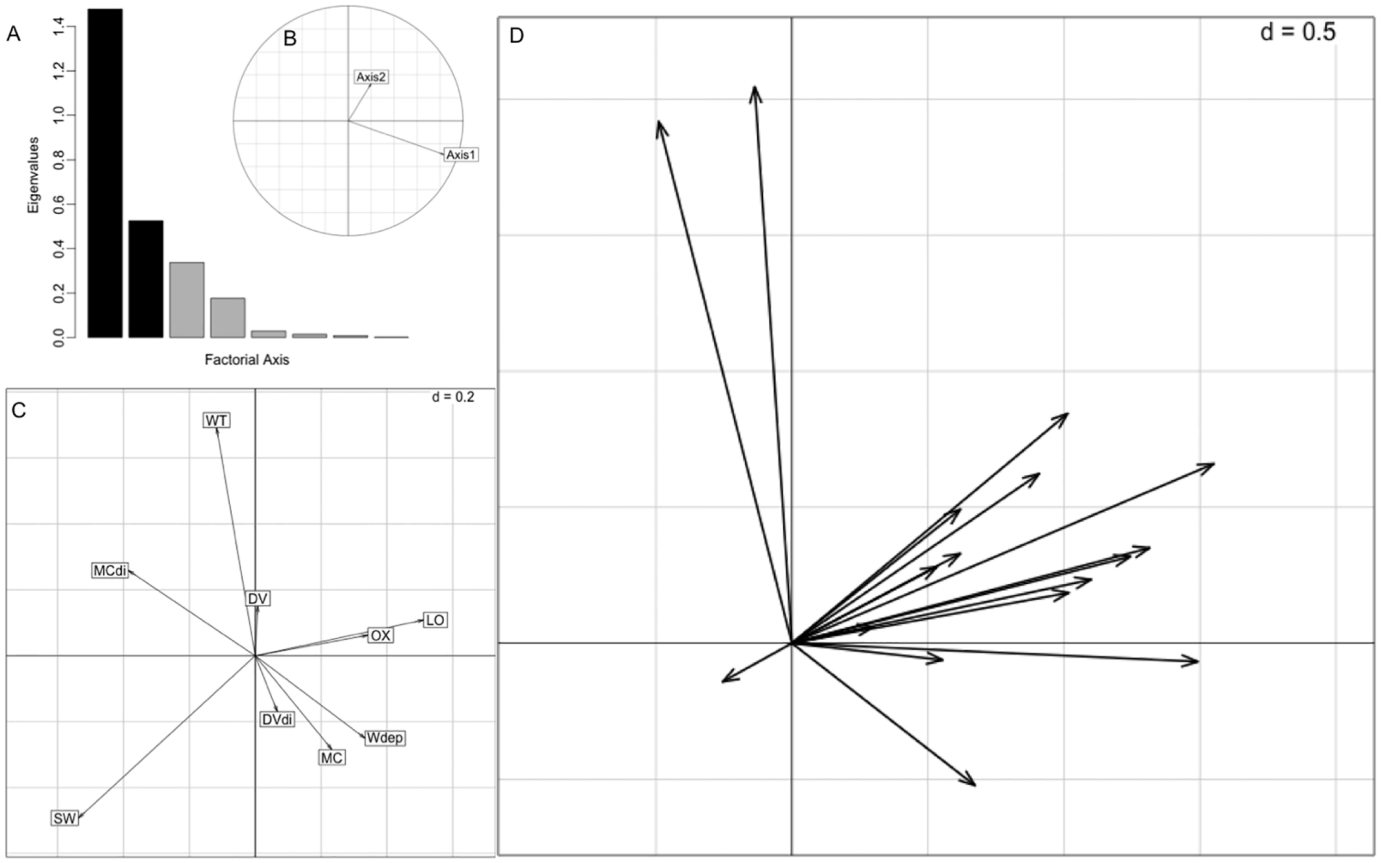
Home range level resource selection plots from the K-select analysis for radio-tracked adult male alligators at Ross Barnett Reservoir and the Pearl River, Mississippi, USA, 2012–2013. (A) Scree plot of eigenvalues where Axis 1 and 2 are shown as black bars and other axes are represented as gray bars. (B) Orthogonality plot for Axis 1 and 2. (C) Habitat variable loadings. Abbreviations of habitat variables: main channel (MC), backwaters and oxbow lakes (OX), floating, emergent vegetation (DV), lotus (LO), forest and swamps (SW), very shallow wetlands (WT), water depth (Wdep), distance to main channel (MCdi), and distance to floating, emergent vegetation (DVdi). (D) Marginality vectors of each animal that point from the centroid of available resource space to the centroid of used resource space.

For selection at the home range scale, the first axis explained 58% of the variation in the data and was related positively to water depth and negatively to distance to main channel (Fig 2A–2C). Inclusion of the second axis accounted for an additional 20% of the variation and corresponded negatively to forests and swamps (Fig 2A–2C). The first eigenvalue λ_1_ was greater than expected (λ_1_ = 1.477, *P* < 0.001) meaning habitat use patterns were informative. Marginality was nonrandom for four animals implying selection or avoidance of habitat variables (Table 1; Fig 2D). Three individuals avoided forests and swamps, one selected for lotus habitat, and one selected for shallow wetlands. Also, two animals selected home ranges near the main channel and reservoir.

**Table 1:** Home range selection marginality values of radio-tracked adult male alligators at Ross Barnett Reservoir and Pearl River, Mississippi, USA, 2012–2013.

Animal	Marginality	Habitat selection scores								
		LO	MC	WT	DV	SW	OX	MCdi	DVdi	Wdep
x0507	2.28	0.89	-0.36	-0.26	0.24	0.49	0.54	-0.57	-0.59	0.21
x0910	2.30	-0.12	-0.36	0.19	0.65	-0.33	0.24	-0.76	-0.78	0.59
x1308	1.54	0.38	-0.36	0.06	0.35	-0.33	0.21	-0.66	-0.72	0.17
x1912	2.12	0.72	-0.15	0.23	0.39	-0.70	0.03	-0.65	-0.69	0.05
x2310	2.46	-0.30	0.30	-0.24	0.33	-0.12	0.13	-0.93	-0.64	0.90
x2509	2.26	0.94	-0.36	-0.10	0.05	-0.60	0.68	-0.42	-0.27	0.41
x2715	2.41	0.65	0.62	-0.12	-0.08	-0.86	0.43	-0.48	0.36	0.54
x2910	1.20	-0.27	-0.36	-0.06	0.39	0.32	-0.21	-0.52	-0.64	0.13
x3090	5.00^a^	-0.30	-0.36	1.77^b^	0.07	-0.96^a^	0.09	0.07	-0.67	-0.50
x3312	3.46^a^	0.05	-0.12	0.02	0.88	-0.61	0.28	-0.92^b^	-0.80	0.86
x3522	7.62^a^	-0.30	-0.36	-0.42	1.47	-0.96^b^	1.53	-0.56	-0.85	0.88
x3915	1.38	0.31	-0.15	0.12	0.51	-0.55	0.18	-0.54	-0.60	-0.05
x5123	3.10	0.98	-0.36	0.17	0.61	-0.83	0.11	-0.57	-0.75	-0.12
x5327	2.38	-0.21	-0.36	-0.36	0.20	-0.11	1.09	-0.51	-0.63	-0.41
x5516	4.19^a^	-0.25	-0.36	1.53	0.24	-0.80	-0.12	0.82	-0.12	-0.50
x5926	1.47	-0.09	0.18	0.02	0.30	-0.27	0.03	-0.78^b^	-0.68	0.43
x6122	3.67	1.30^a^	-0.36	0.01	-0.07	-0.95^a^	0.92	-0.29	0.03	0.10

Randomization tests (n = 10,000 steps) on marginality of each animal.

^a^Significant at 5% level;

^b^Significant at 10% level.

Levels reflect Bonferroni corrections.

Habitats: main channel (MC), backwaters and oxbow lakes (OX), floating, emergent vegetation (DV), lotus (LO), forest and swamps (SW), very shallow wetlands (WT), water depth (Wdep), distance to main channel (MCdi), and distance to floating, emergent vegetation (DVdi).

**Fig 3:**
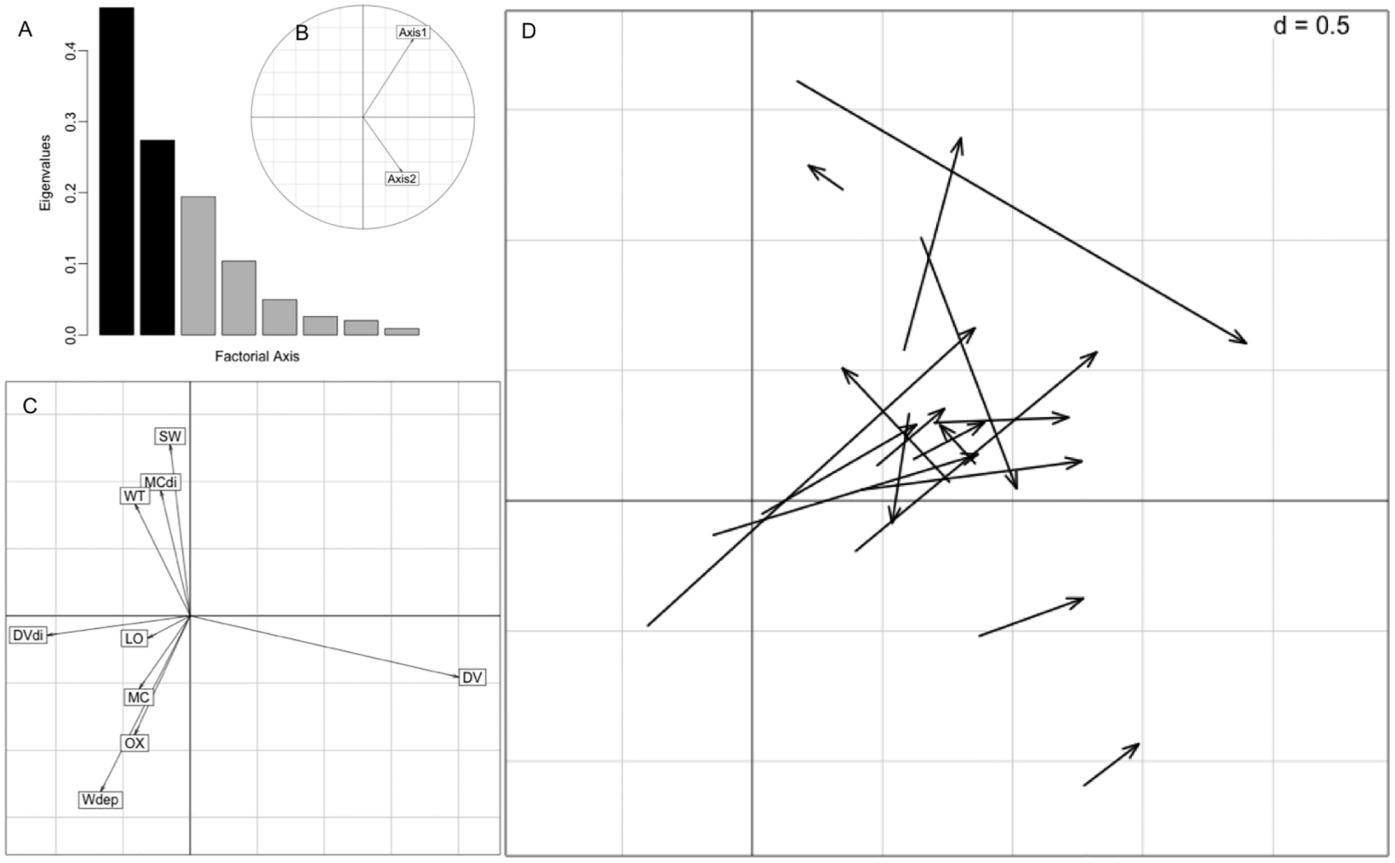
Patch level resource selection plots from the K-select analysis for radio-tracked alligators at Ross Barnett Reservoir and the Pearl River, Mississippi, USA, 2012–2013. (A) Scree plot of eigenvalues where Axis 1 and 2 are shown as black bars and other axes are represented as gray bars. (B) Orthogonality plot for Axis 1 and 2. (C) Habitat variable loadings. Abbreviations of habitat variables: main channel (MC), backwaters and oxbow lakes (OX), floating, emergent vegetation (DV), lotus (LO), forest and swamps (SW), very shallow wetlands (WT), water depth (Wdep), distance to main channel (MCdi), and distance to floating, emergent vegetation (DVdi). (D) Marginality vectors of each animal that point from the centroid of available resource space to the centroid of used resource space.

At the patch scale, the first axis explained 40% of the variation in the data and was related negatively to water depth, main channel, and oxbow habitats (Fig 3A, 3B and 3C). Including the second axis accounted for an additional 15% of the variation and corresponded negatively to shallow wetlands (Fig 3A, 3B and 3C). The first eigenvalue λ_1_ was greater than expected (λ_1_ = 0.460, *P* < 0.001). Marginality was nonrandom for nine animals (Table 2; Fig 3D). Six animals selected for areas with floating emergent vegetation and two animals selected areas near this habitat. Two animals avoided oxbow and backwater areas and two other animals avoided the main channel and reservoir.

**Table 2:** Patch selection marginality values of radio-tracked adult male alligators at Ross Barnett Reservoir and Pearl River, Mississippi, USA, 2012–2013.

Animal	Marginality	Habitat selection scores								
		LO	MC	WT	DV	SW	OX	MCdi	DVdi	Wdep
x0507	1.28^a^	-0.36	0.00	0.25	0.83^a^	-0.01	-0.56^b^	0.00	-0.25	0.10
x0910	0.86^a^	-0.04	0.00	0.02	-0.41	0.73^b^	-0.26	0.08	0.00	0.28
x1308	0.42	-0.22	0.00	-0.10	0.08	-0.02	0.29	0.01	-0.03	0.52
x1912	0.20	-0.23	-0.15	0.04	0.22	0.02	0.14	0.09	-0.05	-0.23
x2310	1.82^a^	0.00	-0.57^b^	0.04	0.61^a^	0.23	-0.20	0.03	-0.18	-1.00^a^
x2509	0.83^a^	0.03	0.00	0.10	0.46	-0.22	-0.33	0.48	-0.29	-0.37
x2715	4.10^a^	-0.17	-0.85^a^	0.61^b^	0.64^a^	0.01	-0.17	0.67^b^	-1.12^a^	-0.91^a^
x2910	2.22^a^	0.03	0.00	0.38	0.58^b^	-1.14^a^	0.09	-0.13	-0.17	0.62^b^
x3090	0.10	0.00	0.00	0.22	-0.13	0.00	-0.11	-0.14	0.06	-0.02
x3312	0.54	-0.23	0.35	-0.10	0.31	0.15	-0.38	-0.15	-0.04	-0.27
x3522	0.13	0.00	0.00	0.00	0.12	0.00	-0.10	-0.05	-0.02	-0.31
x3915	0.51	-0.11	-0.18	-0.31	0.43	0.34	-0.04	-0.19	-0.15	-0.05
x5123	0.15	0.22	0.00	0.05	-0.21	0.00	-0.11	-0.08	0.02	-0.18
x5327	1.43^a^	-0.06	0.00	-0.06	0.10	0.90^a^	-0.75^a^	0.16	-0.09	-0.11
x5516	4.57^a^	0.01	0.00	-1.14^a^	1.45^a^	-0.15	0.08	-0.76^a^	-0.75^b^	0.03
x5926	0.38	-0.05	-0.28	0.26	0.12	-0.09	0.04	-0.18	-0.06	-0.41
x6122	1.42^a^	-0.45	0.00	0.16	0.86^a^	-0.02	-0.38	0.23	-0.41	-0.28

Randomization tests (n = 10,000 steps) on marginality values of each animal.

^a^Significant at 5% level;

^b^Significant at 10% level.

Levels reflect Bonferroni corrections.

Habitats: main channel (MC), backwaters and oxbow lakes (OX), floating, emergent vegetation (DV), lotus (LO), forest and swamps (SW), very shallow wetlands (WT), water depth (Wdep), distance to main channel (MCdi), and distance to floating, emergent vegetation (DVdi).

Alligator TL averaged 2.8 m and ranged from 1.8 to 3.7 m. Multiannual 95% MCP home ranges (mean = 33.0 ± 59.6 ha, range = 0.5–254.8 ha) were not associated with TL (*F*_[1, 15]_ = 0.028, *P* = 0.870, adjusted *R*^2^ = -0.065), even after removing one outlier animal in regards to home range size. We therefore retained all animals for further analyses.

Home range (*F*_[1, 15]_ = 0.208, *P* = 0.655, adjusted *R*^2^ = -0.052) and patch level marginality (*F*_[1, 15]_ = 1.543, *P* = 0.233, adjusted *R*^2^ = 0.033) were not related to body size. Also, mean selection for each habitat was not correlated with body size, except for patch level selection of oxbow habitat (*F*_[1, 15]_ = 4.718, *P* = 0.046, adjusted *R*^2^ = 0.189).

## Discussion

Adult male alligators demonstrated greater selection for habitat at the patch level than at the home range level. As expected, we did not find congruency among selection at the two spatial scales. This suggests habitat selection is hierarchical and that there are fewer limiting factors when selecting a home range where resources are likely abundant. Animals may not select for a particular habitat type at large scales, even if important for specific behaviors (e.g., thermoregulation, foraging), because that habitat may not satisfy other needs [7]. Several adult male alligators exhibited patch level selection for floating emergent vegetation, possibly for thermoregulation, but no individual selected for this habitat at the home range level. Selection at the home range level should reflect consideration of the most limiting factors [7]. At the home range level, adult male alligators avoided swamps and forests, which comprised almost half of the study area. In contrast, some individuals maintained close proximity to the main river channel and reservoir, perhaps as potential foraging sites [26], to improve mobility afforded by the channel [15], and to reduce energetic costs of traversing densely wooded swamps and forested islands [46].

Adult male alligators reportedly select for deep, open water because of availability of large prey and capacity of deep water to buffer extreme temperature fluctuations [21,26,27]. However, in our study no animals selected open water habitats, though two alligators selected home ranges near the main channel and reservoir. In fact, four individuals avoided open water habitats at the patch level and no animal selected for water depth. The relatively shallow water depth in our study area may restrict thermal buffering, thus limiting selection for more preferred areas with deeper water. Alternatively, higher levels of observed recreation and boating activity in open water habitats may constrain alligator use of this habitat. Alligators at RBR were wary when approached, possibly due to hunting, previous capture, and potential risk from recreational boaters, anglers, and hunters. Crocodilians are injured by and generally avoid boats [47,48]. In addition, wave action (e.g., from wind and boat traffic) may impair visual prey location and limit foraging [49].

Unexpectedly, floating, emergent vegetation was the most selected habitat within alligator home ranges (Table 2). This habitat includes stands of dense invasive species (e.g., water hyacinth and alligator weed) that shade out native aquatic plants and may reduce fish habitat quality [40,50]. In addition, dense vegetation can reduce foraging success of aquatic predators due to increased structural complexity [51]. As lesser prey abundance and increased difficulty of prey capture would be expected to reduce foraging suitability of this habitat, we hypothesize alligators may use this dense vegetation as a thermal refuge [26,52]. Behavioral thermoregulation, including basking and use of cooler water, influences alligator daytime movements [17,53,54]. Mat forming vegetation offers shade, likely resulting in cooler water temperatures and providing alligators with a buffer from direct solar radiation.

We found no relationship between space use or resource selection and adult male body size, suggesting size-dependent dominance hierarchies may not influence alligator resource selection or space use in our study area. Other factors, such as density dependence [9], may have a more prominent influence on alligator resource selection. Alligator social behavior has been derived primarily from more homogenous coastal habitats, and captive populations where alligators occur in greater densities compared to riverine systems [21,27,55]. Population density can influence habitat occupation rate and degree of resource competition [3,9,56], with a consequent effect on social dominance structure [57] and habitat selection [58,59]. Body size-dependent social hierarchies may also contribute to population regulation in some crocodilians [60]. Exclusive territories are common in low-density populations [17]. As density increases, defending exclusive territories becomes increasingly difficult and behavioral responses may favor establishment of a strict social system for access to resources, limiting risk of injury or death [17]. Consequently, dominance interactions may be favored in more crowded coastal habitats and captive situations, due to increased social interactions and greater pressures on available resources.

Long-term monitoring suggests the RBR alligator population is increasing perhaps as a result of conservation efforts and other evidence hints that densities may be lower than in coastal systems [27,61]. Therefore, the sampled alligator population may presently be below a density dependent threshold required to saturate available resources and promote the formation of social dominance hierarchies [61]. Given that selection of habitat variables was weak at the home range level, high resource availability could facilitate occupation of suitable habitat by all or most adult males, thus limiting differences in resource selection.

We hypothesized dominance rank was directly proportional to body size based on other studies of crocodilian social behavior [15–17]. However, dominance rank could also be determined by variable behaviors among individual alligators, including temperament [62] and personality [22,53,63]. In addition, more than visual displays of size may influence dominance rank, especially during the breeding season. The role of other behaviors including bellowing and head-slapping in establishing and maintaining dominance is unknown [20]. We did not find a relationship between body size and habitat selection in adult male alligators in our study area, but there may be unaddressed temporal elements to behavior that could affect the relationship. Though alligators may have similar movements and habitat use between day and night [64], at least movements may differ during diurnal and nocturnal periods [20,46,47]. Space use as a function of body size (or dominance rank) may be more detectable at night when interactions among males and foraging are more common. Also, due to low sample size of relocations per animal, we were unable to compare alligator non-breeding to breeding period (April–May) relocations when courtship behaviors may facilitate competition over mates and dominance interactions among competitors.

## Conclusions

Habitat selection of adult male alligators was a scale-dependent process and understanding the mechanisms that influence selection and its relationship to overall fitness [7] may provide information to improve species management and conservation. Aquatic vegetation, water depth, and water temperature of inland riverine systems may be important factors influencing alligator foraging and thermoregulation [26,27,65]. State water management plans and aquatic plant control programs may consider allowing parts of the natural flood plain to flood seasonally for sub-adult dispersal and may consider maintaining patches of thick vegetation to help alligators mitigate their heat budgets. Though apparent habitat suitability and low alligator density may have contributed to the lack of an observed dominance hierarchy, a change in either of these ecological components may increase intraspecific interactions and facilitate a dominance hierarchy. The mechanisms regulating the formation of social hierarchies is an interesting area of behavioral evolutionary ecology that may improve the understanding of animal distribution theory [14,57]. Alligators are generalist top predators with potential top-down effects, mobile vectors of nutrients between terrestrial and aquatic systems, and may function as ecosystem engineers through bioturbation and biodeposition [21,66,67]. Due to these broad and diverse ecological roles, understanding the factors that influence the social dominance and space use of alligators can provide insight into their functional role in the ecosystem.

## Supporting Information

S1 File Habitat categorization map for Ross Barnett Reservoir and Pearl River, Mississippi, USA, 2012–2013. The csv file includes information obtained from the August 2012 United States Department of Agriculture’s National Agriculture Imagery Program, water depths collected by the Mississippi Department of Wildlife, Fisheries, and Parks in August 2005, and plant classifications from Mississippi State University’s Geosystems Research Institute.
(CSV)

S2 File Relocations for radio-tracked adult male alligators at Ross Barnett Reservoir and Pearl River, Mississippi, USA, 2012–2013. The csv file includes animal identification code, location, date and time, and assigned habitat categories determined from S1 File.
(CSV)

S1 Table Total length, number of relocations, and home range size of radio-tracked adult male alligators at Ross Barnett Reservoir and Pearl River, Mississippi, USA, 2012–2013. (DOCX)
