# Supplementary material for: Scale-Dependent Habitat Selection and Size-Based Dominance in Adult Male American Alligators
Source: PLoS One. 2016 Sep 2;11(9):e0161814. doi: 10.1371/journal.pone.0161814 (PMC5010255; doi:10.1371/journal.pone.0161814)
Supplement: S1 Table — (DOCX) [file pone.0161814.s003.docx]

| Animal | Total length (m) | Number of relocations | | | 95% MCP (ha) |
| --- | --- | --- | --- | --- | --- |
|  |  | Year 1 | Year 2 | Combined |  |
| x0507 | 2.388 | 43 | 35 | 78 | 16.3 |
| x0910 | 2.470 | 34 | 40 | 74 | 3.7 |
| x1308 | 2.832 | 25 | 38 | 63 | 9.8 |
| x1912 | 3.569 | 43 | 40 | 83 | 18.3 |
| x2310 | 2.540 | 26 | 31 | 57 | 25.4 |
| x2509 | 2.858 | 35 | 33 | 68 | 46.4 |
| x2715 | 2.734 | 21 | 23 | 44 | 254.8 |
| x2910 | 2.718 | 24 | 34 | 58 | 11.0 |
| x3090 | 2.718 | 16 | 25 | 41 | 5.1 |
| x3312 | 2.607 | 36 | 41 | 77 | 5.7 |
| x3522 | 3.353 | 44 | 41 | 85 | 0.5 |
| x3915 | 3.715 | 40 | 39 | 79 | 36.9 |
| x5123 | 2.426 | 43 | 40 | 83 | 7.9 |
| x5327 | 1.803 | 36 | 39 | 75 | 3.8 |
| x5516 | 2.435 | 36 | 35 | 71 | 60.0 |
| x5926 | 2.692 | 25 | 41 | 66 | 19.3 |
| x6122 | 3.188 | 17 | 26 | 43 | 37.0 |
| Median | 2.718 | 35 | 38 | 71 | 16.3 |

**Table S1. Total length, number of relocations, and home range size of radio-tracked adult male alligators at Ross Barnett Reservoir and Pearl River, Mississippi, USA, 2012–2013.**
